# Convergence, Accommodation, Fusion, and Stereopsis: What Keeps the Eyes Aligned in Intermittent Exotropia?

**DOI:** 10.1155/2018/9546979

**Published:** 2018-07-26

**Authors:** Costantino Schiavi, Valentina Di Croce, Laura Primavera, Filippo Tassi

**Affiliations:** Department of Experimental, Diagnostic, and Specialty Medicine (DIMES), Ophthalmology Service, St. Orsola-Malpighi Teaching Hospital, University of Bologna, Via P. Palagi 9, 40138 Bologna, Italy

## Abstract

**Purpose:**

To investigate the relationships between angle of deviation, fusional convergence and divergence amplitude, AC/A ratio, near point of convergence (NPC), and myopia-phoria in intermittent exotropia (IXT).

**Methods:**

93 patients with IXT, divided into two groups, were recruited in the study. In Group A (73 patients), fusional convergence and divergence amplitudes, NPC, and AC/A ratio were studied and compared with a control group. In Group B (in 20 patients), myopia-phoria while switching from monocular to binocular view was studied with an infrared video retinoscopy and compared with a control group.

**Results:**

In Group A, positive fusional amplitudes, NPC, and AC/A ratio of IXT patients did not differ from those of normals. Negative fusional amplitudes were significantly higher in the patient group than in the control group. In Group B, myopic shift was statistically significantly higher in the patient group and there was a statistically significant positive correlation between myopic shift and angle of strabismus.

**Conclusions:**

Apart from the role of fusional convergence which accounts for myopia-phoria, that of the other binocular functions in the control at near of IXT and conversely their specific role in the pathogenesis of IXT remain unclear and the cause of divergent infantile strabismus is still unknown.

## 1. Introduction

Exotropia (XT) or divergent squint usually affects about 1% of the general population and it occurs more commonly in the Middle East, subequatorial Africa, and the Orient than in the United States [1]. XT may result from certain obstacles to development or maintenance of binocular vision or may be secondary to the defective action of the medial rectus muscles. However, in most cases of XT of infantile origin, neither binocular vision is impaired, nor the function of the medial rectus muscles is altered, at least at an early stage. The role of innervational factors like convergence and divergence and that of accommodation, stereopsis, and motor and sensory fusion in the etiopathogenesis of infantile exotropia have long been the subject of debate among ophthalmologists, and in essence, the cause of concomitant infantile XT is still unknown.

In terms of level of control of the deviation, exodeviations may be distinguished into exophoria (XPH), intermittent exotropia (IXT), and exotropia (XT). The most common type of XT is IXT, a condition in which periods of manifest exodeviation, usually at distance fixation, alternate with periods of binocular alignment, commonly at near fixation. IXT comprises about 75–90% of the cases of exotropia and may be preceded by a stage of XPH [1, 2].

In terms of amount of the deviation at near and distance fixation, XT has been distinguished into four patterns (*basic*, *convergence insufficiency pattern*, *divergence excess pattern*, *and simulated divergence excess pattern*), according to the classification proposed by Duane [1]. This classification relies on Duane's concepts of *fusional convergence insufficiency* and *fusional divergence excess* and is based upon measurements of the distance and near deviation. These concepts have proved to be misleading, because at present no scientific evidence exists for excessive tonic divergence innervation in XT and convergence insufficiency pattern exodeviations not necessarily have a distant near point of convergence (NPC). However, Duane's classification maintains a clinical and practical utility.

The sensory status in IXT is double as normal binocular vision alternates with suppression of the deviated eye. When the eyes are aligned, usually at near fixation, patients show normal binocular vision and stereopsis. On the other hand, in the phases where the deviation is manifest, that is, at distance fixation, suppression takes place [3]. It is not clear if the intermittent deviation induces intermittent suppression of the deviated eye or if the opposite happens, that is, suppression induces the deviation.

The role of sensory and motor fusion, stereopsis, convergence, and accommodation in the control of the deviation, and conversely their weight in the pathogenesis of concomitant congenital XT is still debated. Recently, a study regarding a wide number of IXT patients found no strong associations between stereopsis, amount of strabismus, and control of the deviation [4].

It is generally believed that in IXT patients, tonic convergence and accommodation through accommodative convergence contribute to maintain ocular alignment at near fixation [5]. The accommodative convergence-to-accommodation (AC/A) ratio has been supposed to be involved in the control of the deviation at near fixation in IXT. However, not necessarily IXT patients who can control the deviation at near exhibit a high AC/A ratio.

Instead, in IXT patients, the so-called phoria-myopia response occurs at near fixation [6, 7]. Phoria-myopia is described as a myopic shift at near fixation due to the contraction of the ciliary muscle while switching from monocular to binocular fixation. A possible explanation of this myopic shift during the control of the deviation at near is the stimulation of accommodation elicited by fusional convergence excessively stressed in the effort of overlap with the exodeviation [6, 8]. Accommodation and convergence are interdependent functions, and normal to a certain degree of accommodation corresponds to a certain amount of convergence and vice versa. Interdependence between accommodation and convergence is expressed by the accommodative convergence-to-accommodation (AC/A) and the convergence accommodation-to-convergence (CA/C) ratios.

In patients with IXT, a greater effort to converge is required to fixate a near target binocularly compared with patients with orthophoria. It is debated if in IXT the control of the deviation is mainly warranted by accommodation through the AC/A ratio or by fusional vergence through the CA/C ratio.

Fusional vergence or motor fusion is an optomotor reflex that produces corrective eye movements to overcome retinal image disparity. Fusional vergence is classified according to the plane of eye movements, and the maximum amount of eye movement produced is referred to as amplitude [9]. IXT patients are reported to have high convergence fusional amplitudes that they use to compensate the deviation [10]. Nevertheless, this particular clinical meaning was never studied in deep. Moreover, it is quite unclear if there is a connection between fusional amplitudes and control of the deviation. It is not easy to objectively quantify the degree of control of IXT during a single ocular examination. Scoring scales have been proposed in an attempt to better assess the control of exodeviation [11].

The control of the deviation is commonly indirectly deduced during the follow-up from signs like degraded stereopsis, onset of amblyopia, increased deviation, or symptoms like asthenopia, visual discomfort, and diplopia, even if in IXT suppression usually protects from diplopia.

To investigate the relationships between angle of deviation, fusional convergence and divergence amplitudes, AC/A ratio, NPC, and myopia-phoria in IXT, we studied a series of patients referred to our department in the last five years.

## 2. Materials and Methods

Ninety-three patients with IXT were recruited in the study. All the patients were visited the Ophthalmology Unit of the Department of Experimental, Diagnostic and Specialty Medicine (DIMES) of St. Orsola-Malpighi Hospital in Bologna. Patients were included in the study if they were able to fully participate in the examination. The exclusion criteria were lack of cooperation, previous strabismus surgery, amblyopia, absence of random-dot stereopsis, developmental delay or sensory or paralytic exotropia, major systemic diseases, and other ophthalmologic disorders such as retinal and optical nerve diseases.

Visual acuity was required to be +0.20 LogMAR or better in each eye for inclusion. All the patients enrolled in the study presented at least 240” random-dot stereopsis at near at the TNO test.

Patients were divided into two groups (Groups A and B) based on the kind of evaluation made. All the types of XT were represented, according Duane's classification. Patients or patients' parents if underage gave informed consent after the purpose of this study was explained, and the procedures conformed to the tenets of the Declaration of Helsinki.

### 2.1. Group A

Seventy-three patients from 3 to 64 years of age (average 17.77 ± 16.02 years) with IXT were studied as regards angle of deviation, fusional convergence and divergence amplitudes (break point and recovery point), and NPC. Twenty orthophoric subjects aged 13 to 25 years (average 19.05 ± 2.72 years) served as controls.

All clinical tests were performed with full refractive correction when required. The angle of deviation was assessed using prism and alternate cover testing at distance (3 meters) and then at near (33 cm) and recorded in prism diopters (PD).

Fusional convergence amplitudes were studied using a 1 to 40 PD fixed horizontal prism bar (Beren's). An accommodative target was used first at distance (3 meters) and then at near (33 cm).

Base-in prism for divergence and base-out prism for convergence were used. The fusional convergence break point was identified as the base-out prism strength at which an exotropia occurred and could not be compensated. A short time was allowed for spontaneous recovery of fusion after an exotropia occurred. Afterwards, the prism strength was gradually decreased until the patient could fuse the induced exotropia. When fusion occurred, the value of the prism strength was recorded as recovery point.

In IXT children, convergence break point and recovery point were often confirmed by brief cover test because they could lack awareness of diplopia.

Total negative fusional amplitude at near was defined as the sum of the angle of deviation at near plus negative fusional amplitude at near; total negative fusional amplitude at distance was defined as the sum of the angle of deviation at distance plus negative fusional amplitude at distance. Convergence fusional reserve was defined as the additional convergence fusion, superimposed on ability to fuse the exodeviation, measured by determining the fusion break point [10].

Fusional divergence amplitude was measured with base-in prisms of increasing power starting from the base-in prism totally correcting the exodeviation. Break point and recovery point of fusional divergence amplitude were recorded. Near stereopsis was evaluated with the TNO test (Laméris Ootech B.V., Nieuwegein, the Netherlands).

The near point of convergence (NPC) was evaluated objectively. Objective NPC was measured using an accommodative target moving in Beren's ruler. The examiner moved the target toward the patient's eye and stopped it when a deviation of one eye was observed; the distance from this point to the lateral canthus was measured. NPC was repeated twice for all patients and subjects, and the two measures were averaged.

The control group underwent the same evaluation and analysis.

### 2.2. Group B

Myopic shift under binocular viewing conditions (phoria-myopia) and pupil diameter changes were studied in 20 IXT patients and 21 orthophoric subjects considered as control group with the PlusoptiX A12R infrared video retinoscopy (PlusoptiX A12R, Nürnberg, Germany). In both groups, the AC/A ratio was measured with the gradient method.

Exclusion criteria for the infrared video retinoscopy were pupil size below 4 mm and above 8 mm and/or refractive errors beyond the limits of the PlusoptiX A12R (i.e., −7.0/+5.0 diopters) in either eye.

The age of the patients ranged from 4 to 26 years of age and averaged 11.75 ± 6.05 years. The age of the controls ranged from 16 to 32 years and averaged 25.38 ± 4.11 years.

In the patients group, the far deviation and the distance deviation ranged from −50 to −2 PD (average: −20 ± 13.38) and from −55 to −4 PD (average: −20.85 ± 13.99), respectively.

The angle of strabismus was assessed using the alternate prism and cover test at distance (3 meters) and then at near (33 cm) fixation and recorded in PD.

Refraction and pupillary diameter were measured simultaneously with the PlusoptiX A12R. PlusoptiX A12R measures both eyes simultaneously in 0.5 seconds at a distance of one meter. Therefore, even small children with a short attention span can be measured.

Measurements were made under both monocular and binocular viewing conditions, without spectacle correction. To obtain accurate measurements, all the patients have been set under mesopic light conditions. The simultaneous measurement of both eyes enabled a reliable refraction assessment and pupil size evaluation during binocular view. For the monocular condition, each patient covered one eye with his own hand and the refraction and pupil size were measured for one eye at a time.

The target distance was set at 1 m, and the results of three measurements were averaged. Refractive changes while switching from monocular to binocular viewing conditions were recorded. AC/A ratio was measured with the prism and alternate cover test with the full optical correction for the patients who needed lens, and then the deviation was measured again at the same distance of 1 meter with −3.00 D lenses placed before the spectacles. The stimulus AC/A ratio was calculated by dividing the difference in the deviation as detected in the two conditions by the accommodative demand of −3.00 D (far gradient method).

The control group underwent the same evaluation and analysis.

## 3. Statistical Analysis

### 3.1. Group A

Fusional amplitude (FA), break point, and recovery point of IXT patients were compared to those measured in a control group of orthophoric subjects using the Mann–Whitney *U* test. Near point of convergence (NPC) was also compared between the two groups. Statistical analyses were performed using SPSS (SPSS, Chicago, Illinois, USA), and *p* value < 0.05 was considered statistically significant.

### 3.2. Group B

Statistical analyses were performed using SPSS (SPSS, Chicago, Illinois, USA).

Comparisons between binocular and monocular refraction, AC/A ratio, and pupillary diameter in the patient group and the control group were statistically analyzed using the Mann–Whitney *U* test and Spearman's correlation test. *P* value < 0.05 was considered statistically significant.

## 4. Results

### 4.1. Group A

Statistical data are summarized in Table 1. The median angle of deviation was 18.16 PD (range 8 to 55 PD ± 9.73 sd) at distance and 17.6 PD (range 8 to 50 PD;  ±10.01 sd) at near.

Positive fusional amplitudes in the patient group were 23.15 ± 9.66 PD at near and 17.05 ± 9.39 PD at far, while positive fusional amplitudes in the control group were 24.6 ± 9.94 PD at near and 17.8 ± 6.61 PD at far. There was no statistically significant difference between patient and control groups for positive fusional amplitude (distance: *p*=0.184; near: *p*=0.641) and positive recovery point (distance: *p*=0.158; near: *p*=0.469) at near and distance. There was also no statistically significant difference between the two groups for NPC (*p*=0.160). Negative fusional amplitudes at distance and near were statistically significantly higher in the patient group than in the control group (distance: 12.22 ± 6.62 PD versus 6.30 ± 1.62 PD, *p* < 0.0001; near: 15.55 ± 5.11 PD versus 12.00 ± 3.37 PD, *p*=0.003).

Difference between negative break point and recovery point was found to be statistically significantly higher in the patient group than in the control group (distance: 9.90 ± 6.14 PD versus 3.80 ± 1.58 PD, *p* < 0.0001; near: 12.56 ± 4.67 PD versus 8.70 ± 3.19 PD, *p* < 0.0001).

### 4.2. Group B

Statistical data are summarized in Table 2. The mean angle of deviation was −20 ± 13.38 PD (range: −2/−50 PD) at distance and −20.85 ± 13.99 PD (range: −6/−55 PD) at near.

In patients, the spherical refractive error ranged from −4.75 to + 1.75 diopters (D) (average: −0.03 ± 1.5 D) under monocular viewing conditions and from −4.75 to + 1.75 D (average: −0.45 ± 1.53 D) under binocular viewing conditions. In the control group, the spherical equivalent refractive error ranged from −4 to −0.12 diopters (D) (average: −1.57 ± 1.35 D) under monocular viewing conditions and from −4. to −0.12 D (average: −1.59 ± 1.36 D) under binocular viewing conditions.

The mean value of the myopic shift in the patients was 0.44 ± 0.42 D.

There was statistically significant difference between patients and controls in myopic shift (*p* < 0.0001) while switching from monocular to binocular viewing conditions.

An intragroup analysis in the patient group revealed no statistically significant difference in myopic shift between patients aged over 16 years (5 patients) and patients aged under 16 years (*p*=0.328). A statistically significant difference in pupillary diameter between patients and controls was found in either monocular (4.75 ± 0.80 mm versus 5.57 ± 0.79 mm, *p*=0.027) or binocular viewing conditions (5.6 ± 1.01 mm versus 6.13 ± 0.88 mm, *p*=0.013).

The changes in pupillary diameter when switching from monocular to binocular viewing conditions did not statistically significantly differ between patients and controls (*p*=0.166).

In the patient group, there was a statistically significant correlation between myopic shift and the angle of strabismus at either distance (*r*=0.58, *p*=0.007) or near fixation (*r*=0.56, *p*=0.009) (Table 3).

AC/A mean value was 5.61 ± 2.68 in the patient group and 6.12 ± 0.47 in the control group. This difference was not statistically significantly different (*p*=0.296).

## 5. Discussion

What keeps the eyes aligned in IXT is still a debated question. First of all, the role of convergence fusional amplitudes in IXT is controversial. They are not defective in IXT patients, as can be expected; rather, they may be wider in IXT patients than in orthophoric subjects. Nevertheless, they may contribute to compensate the deviation only intermittently, more at near than at distance fixation. Very often large intermittent exodeviations go along with wide convergence fusional amplitudes, and these latter seem to chase the deviation, in the sense that the larger the deviation, the wider the convergence fusional amplitude. So, among the causes of IXT, a supposed insufficiency of convergence fusional amplitude cannot be enlisted. On the other hand, the surprising finding of increased divergence fusional amplitudes in IXT patients may be probably explained by the underestimation of the angle of strabismus during the examination. Regarding a possible role of the NPC in the pathogenesis of XT, this can be excluded, as in XT patients the NPC is very often normal, even in the convergence insufficiency pattern XT. To try to explain the control of the deviation at near, it has long been assumed that accommodation through the AC/A ratio plays the main role in compensating the near deviation in IXT. High AC/A ratios have been supposed to make this control possible. According to this assumption, accommodation drives convergence during the control of distance exotropia for near fixation. Likewise, overminus lens treatment in clinical practice is believed to work by stimulating accommodation and through accommodation, accommodative convergence, thus favoring the control of the deviation [12]. However, this hypothesis does not explain why the deviation may be compensated also in cases of normal or even low AC/A ratios. Moreover, the clinical observation of a myopic shift when switching from monocular to binocular fixation during the control of distance exotropia for near fixation in IXT patients gave rise to the hypothesis that a role in the control of the deviation may be played by fusional convergence, through the CA/C ratio [13, 14]. This myopic shift has been called “phoria-myopia” [6, 7] and has been described to be detectable only in adults and not in children with IXT [15]. In the present study, we objectively assessed the refractive error and the pupillary diameter under either monocular or binocular viewing conditions with a handheld infrared photoscreener in a series of IXT patients whose AC/A ratio measured with the gradient method did not statistically significantly differ from that of controls. Patient series included children, teenagers, and adults. In this study, a significant myopic shift under binocular conditions was detected in IXT patients aged 4 to 26 years without differences between children and adults. Phoria-myopia can occur indeed also in children with IXT, and increased vergence demand to control intermittent distance exotropia for near is likely the main cause of overaccommodation leading to the myopic shift. Therefore, one may argue that overminus lens therapy may work by correcting the myopic shift caused by overaccommodation driven by fusional convergence, rather than by inducing blur-driven accommodative convergence. The results of our study confirm those of other authors. It is not necessarily a high AC/A ratio that allows to control the deviation at near fixation in distance IXT, by determining overconvergence through accommodation (accommodative convergence). Accommodation in such conditions is likely to be driven by fusional convergence stimulated by crossed disparity [16]. The latter drives accommodation through the CA/C ratio. So, accommodation in phoria-myopia seems to be a consequence of convergence and not the result of a reaction to blur for near fixation. It is likely that in IXT patients, an additional convergence is required to control the deviation for near. This induces greater accommodation than in orthophoric subjects, with greater accommodation being induced in larger deviations. However, the individual accommodative response varies greatly among IXT patients, depending not only on the angle or the type of deviation, but probably on the CA/C ratio [13, 14, 17]. CA/C ratio, which is difficult to measure and is not routinely assessed in the clinical practice, could explain some common clinical findings, like the therapeutic effect of overminus lenses on the control of the deviation and myopia progression in IXT patients [12, 18]. Overminus lenses facilitate fusional convergence by correcting the myopic shift, thus making the control of the deviation easier. On the other hand, it may be presumed that a high CA/C ratio may favor myopia progression by inducing an excessive accommodative stress at near fixation in children and teenagers with IXT. Indeed, the incidence of myopia in IXT patients at age 20 years has been reported to be very high [19]. Main limits of the present study are the small sample size, the presence in the sample of various types of intermittent exotropia, and the difficulty in calculating the AC/A ratio mostly in children and in classifying the patients into conventional clinical groupings.

Finally, it remains to be established how suppression interferes with XT, mostly IXT. Suppression of the input from the deviated eye takes over binocular vision when one eye is misaligned and prevents the patient from diplopia. Suppression in IXT is supposed to be a consequence of the deviation occurring at an early age and it is thought to be a sensory compensatory adaptation to an infantile deviation. In IXT, suppression alternates with normal binocular vision which is present when the eyes are aligned. So, in IXT also suppression is intermittent, like the deviation. Whether suppression of the input from one eye follows or rather precedes the deviation of that eye being somehow a cause or the main cause of the divergent strabismus, it has not yet been established and it is not easy to demonstrate.

## 6. Conclusions

In IXT, the NPC is not farther than that in normals, convergence fusional amplitudes are not defective, and the AC/A ratio is not necessarily high. The increased negative fusional vergences may be the result of defective quantifying of the actual amount of the deviation. Phoria-myopia which depends on the convergence accommodation-to-convergence (CA/C) ratio occurs in children and adults with IXT, and increased vergence demand to control intermittent distance exotropia for near is likely the main cause of overaccommodation leading to the myopic shift. In conclusion, apart from the role of fusional convergence which accounts for myopia-phoria, that of the other binocular functions in the control at near of IXT and conversely their specific role in the pathogenesis of IXT remain unclear and the cause of divergent infantile strabismus is still unknown.

## Figures and Tables

**Table 1:** Group A: descriptive statistics for patient group and control group (Mann–Whitney *U* test).

	Patient group				Control group				*U* test
	Min	Max	Mean	Std. deviation	Min	Max	Mean	Std. deviation	*p* value
Age	3.00	64.00	17.77	16.02	13.00	25.00	19.05	2.72	NE
Angle at far	8.00	55.00	18.16	9.73	NE	NE	NE	NE	NE
Angle at near	8.00	50.00	17.60	10.01	NE	NE	NE	NE	NE
Positive FA at near	8.00	45.00	23.15	9.66	10.00	45.00	24.60	9.94	0.64
Positive FA at far	6.00	45.00	17.05	9.39	10.00	40.00	17.80	6.61	0.18
Positive FA at near recovery	2.00	40.00	17.32	8.65	4.00	40.00	18.60	8.20	0.47
Positive FA at far recovery	2.00	40.00	11.93	8.34	6.00	30.00	13.30	6.52	0.16
Positive reserve at near	−29.00	37.00	5.55	12.33	NE	NE	NE	NE	NE
Negative FA at near	6.00	30.00	15.55	5.11	4.00	18.00	12.00	3.37	0.00
Negative FA at far	4.00	30.00	12.22	6.62	4.00	8.00	6.30	1.63	0.00
Negative FA at near recovery	2.00	25.00	12.56	4.67	2.00	14.00	8.70	3.20	0.00
Negative FA at far recovery	2.00	25.00	9.90	6.14	2.00	6.00	3.80	1.58	0.00
Total negative FA at near	12.00	70.00	30.16	12.81	NE	NE	NE	NE	NE
Total negative FA at far	12.00	75.00	30.38	14.84	NE	NE	NE	NE	NE
NPC	8.00	33.00	16.37	7.68	8.00	23.00	12.65	4.09	0.14

FA = fusional amplitude; NE = not evaluable

**Table 2:** Group B: descriptive statistics for patient group and control group (Mann–Whitney *U* test).

	Patients				Controls				*U* test	
	Min	Max	Mean	Std. deviation	Min	Max	Mean	Std. deviation	*p* value	
Age	4.00	26.00	11.75	6.21	16.00	32.00	25.38	4.21	NE	
AC/A ratio	1.80	10.60	5.62	2.68	5.40	6.90	6.12	0.47	0.30^(1)^	
Mean myopic shift	0.00	1.75	0.44	0.42	−0.63	0.13	−0.05	0.15	0.33^(2)^	0.00^(3)^
Mean pupil binocular	3.80	6.80	4.75	0.81	3.70	7.05	5.59	1.03	0.01^(4)^	0.17^(5)^
Mean pupil monocular	4.40	7.80	5.58	0.79	4.45	7.60	6.14	0.88	0.03^(6)^	
Angle at far	−50.00	−2.00	−20.00	13.73	NE	NE	NE	NE	NE	
Angle at near	−55.00	20.00	−18.85	17.02	NE	NE	NE	NE	NE	
SE monocular	−4.37	1.87	−0.13	1.43	−4.00	−0.125	−1.57	1.35	NE	
SE binocular	−4.62	1.62	−0.55	1.44	−4.00	−0.125	−1.59	1.36	NE	

Test for (1) AC/A ratio: comparison between patient and control groups; (2) myopic shift in the patient group: comparison between adults and children; (3) myopic shift: comparison between patient and control groups; (4) changes in pupillary diameter in binocular viewing condition: comparison between patient and control groups; (5) pupillary diameter changes during switch from monocular to binocular view condition: comparison between patient and control groups; (6) changes in pupillary diameter in monocular viewing condition: comparison between patient and control groups. NE = not evaluable

**Table 3:** Group B: Pearson correlation.

		Age	Angle at far	Angle at near	Mean myopic shift	AC/A	Mean pupil shift
Age	Pearson correlation	1	—	—	—	—	—
	Sig. (2-tailed)	—	—	—	—	—	—
Angle at far	Pearson correlation	−0.38	1	—	—	—	—
	Sig. (2-tailed)	0.09	—	—	—	—	—
Angle at near	Pearson correlation	−0.55	0.66	1	—	—	—
	Sig. (2-tailed)	0.01	0	—	—	—	—
Mean myopic shift	Pearson correlation	0.33	−0.58	−0.57	1	—	—
	Sig. (2-tailed)	0.15	0.01	0.01	—	—	—
AC/A	Pearson correlation	−0.18	−0.31	0.33	−0.01	1	—
	Sig. (2-tailed)	0.45	0.18	0.16	0.74	—	—
Mean pupil shift	Pearson correlation	0.25	−0.42	−0.17	0.1	0.27	1
	Sig. (2-tailed)	0.29	0.07	0.47	0.69	0.24	—

## Data Availability

All data used to support the findings of this study are included within the article.
